# Influence of Femtosecond Laser Surface Nanotexturing on the Friction Behavior of Silicon Sliding Against PTFE

**DOI:** 10.3390/nano9091237

**Published:** 2019-08-30

**Authors:** Isabel Alves-Lopes, Amélia Almeida, Vítor Oliveira, Rui Vilar

**Affiliations:** 1 CeFEMA–Center of Physics and Engineering of Advanced Materials, Instituto Superior Técnico, Universidade de Lisboa, Avenida Rovisco Pais, 1049-001 Lisboa, Portugal; 2 Instituto Superior de Engenharia de Lisboa, Avenida Conselheiro Emídio Navarro No. 1, 1959-007 Lisboa, Portugal

**Keywords:** laser surface texturing, laser-induced periodic surface structures, LIPSS, silicon, PTFE, friction

## Abstract

The aim of the present work was to investigate the influence of laser-induced periodic surface structures (LIPSS) produced by femtosecond laser on the friction behavior of silicon sliding on polytetrafluoroethylene (PTFE) in unlubricated conditions. Tribological tests were performed on polished and textured samples in air using a ball-on-flat nanotribometer, in order to evaluate the friction coefficient of polished and textured silicon samples, parallel and perpendicularly to the LIPSS orientation. In the polished specimens, the friction coefficient decreases with testing time at 5 mN, while it increases slightly at 25 mN. It also decreases with increasing applied load. For the textured specimens, the friction coefficient tends to decrease with testing time in both sliding directions studied. In the parallel sliding direction, the friction coefficient decreases with increasing load, attaining values similar to those measured for the polished specimen, while it is independent of the applied load in the perpendicular sliding direction, exhibiting values lower than in the two other cases. These results can be explained by variations in the main contributions to friction and in the wear mechanisms. The influence of the temperature increase at the interface and the consequent changes in the crystalline phases of PTFE are also considered.

## 1. Introduction

Laser surface texturing is one of the most versatile methods for controlling the tribological properties of materials, since it allows creating a variety of surface textures at the micro and nanoscales in a wide range of materials, with excellent control of the surface features shape and size and negligible degradation of the bulk material [1]. Femtosecond lasers are particularly promising for this application because, due to their high intensity and extremely short interaction time, they significantly reduce undesirable thermal effects, leading to better control of the surface topography, improved accuracy, better reproducibility and less surface contamination [2,3,4]. Furthermore, due to the non-linear nature of the laser-material interaction mechanisms for these lasers, wide band gap transparent materials, as well as metals and semiconductors, can be easily processed [5].

When surfaces are irradiated with femtosecond laser pulses at fluences slightly above the material ablation threshold, parallel surface undulations known as laser-induced periodic surface structures (LIPSS) are formed on the surface of a wide variety of materials [6,7,8,9]. The surface topographies by femtosecond laser treatment were previously described by Bonse et al. [9] and Oliveira et al. [10,11,12]. In this paper, we consider LIPSS with a periodicity of the same order of magnitude of the radiation wavelength, known as low-spatial frequency LIPSS (LSFL). The formation of LSFL is due to a periodic modulation of the absorbed radiation intensity that arises as a consequence of the interference between the incident laser beam and electromagnetic waves propagating parallel to the surface [13,14]. This modulation of the laser absorbed energy generates a periodic variation of the material surface temperature and, consequently, of the transformations that occur in the material, leading to the imprinting of the surface topography by differential ablation [15]. LIPSS affect surface properties such as wettability [16], light reflectivity and transmittance [17,18,19], cell behavior and osseointegration capability of implant materials [16,20], bacteria adhesion and biofilm formation [21], as well as the materials tribological behavior, in particular its friction coefficient [6,22,23,24,25,26,27,28].

The studies available of the influence of LIPSS on the tribological behavior of materials reveal often a complex behavior [22,23,24,25,26,27,28,29,30,31]. The tribology of polytetrafluoroethylene (PTFE) has been extensively investigated (e.g., [32,33,34,35,36,37,38]), but the influence of surface nanotextures on this behavior remains poorly understood. He et al. [39] studied the effect of textures consisting of square section pillars and grooves of different dimensions on the friction of a poly(dimethylsiloxane) (PDMS) elastomer using nanoscratch testing. The tests were conducted at room temperature, using a 1.6 mm diameter 304 stainless-steel bearing ball, and a Rockwell indenter diamond tip with 25 µm radius, respectively, 5, 10 and 25 mN applied loads and 1 µm/s sliding speed. The authors found that the square pillar textures significantly reduce the macroscale friction coefficient, an effect explained by the reduction of the real contact area, and, consequently, of the adhesion contribution to friction, as compared to flat surfaces. A linear relationship between the friction coefficient and (load)^−1/3^ was observed for the textured specimens, as predicted in classical friction theory for elastic contacts, but this relationship did not hold for the flat surfaces. Analysis of the experimental data using the Johnson–Kendall–Roberts theory [40], showed that this difference in behavior is due to a larger adhesion force in the case of the flat specimens, confirming the explanation provided for the effect of surface texturing on friction. At the microscale, the influence of texture on the friction coefficient is less noticeable, particularly at 25 mN load, because the reduction of adhesion is offset by the effect of mechanical interlocking due to the comparable size of the diamond tip and the surface features dimensions. In the case of the grooved textures, the friction coefficient is anisotropic, being largely controlled by the stick-slip behavior of the system.

The silicon-PTFE tribosystem is important for a wide range of applications, particularly in micro-electro-mechanical systems (MEMS). The use of polymers in microsystems has been increasing, as replacements of silicon in some parts (e.g., [41]), as coatings and lubricants (e.g., [42,43,44]) or in hybrid microdevices containing silicon electronics [45], and PTFE is one of the most attractive polymers for these applications due to its low dielectric constant, low surface adhesion, chemical inertness, electrical and thermal insulation properties and self-lubricating characteristics [46,47,48,49], so the study of the friction behavior of the silicon/PTFE system at the nanoscale is of utmost importance. In the present work, we studied the influence of surface textures produced by femtosecond laser on the friction behavior of single crystalline silicon sliding on PTFE, under unlubricated conditions.

## 2. Materials and Methods

<111> single crystal wafers of p-doped silicon with 525 ± 25 µm thickness were cut into approximately 1 cm × 1.5 cm samples and cleaned in an ultrasonic bath with acetone followed by isopropanol. Surface texturing was performed by the direct writing technique, using a Yb:KYW chirped-pulse regenerative amplification laser system (Amplitude Systèmes s-Pulse HP, Pessac, France) with a central wavelength of 1030 nm, pulse duration at full width at half maximum (FWHM) of 560 fs and a Gaussian intensity distribution in the cross-section of the beam, with a (1/e^2^) radius of $\omega_{0}\sim 50\;µm$ at the sample plane, calculated using the method of Liu [50]. The laser beam was focused by a 10 mm focal length lens on a plane 12 mm above the surface of the specimen, leading to a spot size of about 100 µm. The polarization direction of the linearly polarized laser beam was controlled by a half-wave (λ/2) plate. The laser treatment was performed by moving the samples under the stationary beam using a computer-controlled XYZ stage. In order to cover all the surface with a uniform LIPSS structure, scanning of the sample is performed by moving the stage in the XX direction under the stationary beam. After completing one track, the sample is displaced in the YY direction and then scanned in the XX direction again. The XX direction corresponds to the direction of the laser beam polarization, thus generating LIPSS perpendicular to the laser tracks. Consecutive tracks were partially overlapped, in the YY direction, by moving the specimen about 50% of the width of a single laser track in the XX direction. This allows texturing the entirety of the sample’s surface with uniform LIPSS. The laser treatments were performed with an average pulse energy of 100 µJ, calculated from the average laser beam power measured with an Ophir Photonics 10A-SH-V1.1RoHS powermeter (Andover, MA, USA). Taking into consideration that the laser spot diameter at the specimens’ surface was 200 µm, the average fluence was 0.32 J cm^−2^. The scanning speed was 1 mm/s and the pulse frequency 200 Hz, leading to an average of 40 pulses per surface spot.

The textured surfaces were characterized using a LEICA DM 5500B binocular optical microscope (Wetzlar, Germany) and a JEOL JSM 7001F field emission gun scanning electron microscope (FEG-SEM, Akishima, Tokyo, Japan) operated in the secondary electrons imaging mode. The surface texture was quantitatively characterized by analyzing stereoscopic pairs of SEM images of the same areas using Alicona-MeX software (Graz, Austria).

The tribological tests were performed in a ball-on-flat linear reciprocating sliding configuration, using a CSM Instruments NTR1 nanotribometer and 3 mm diameter PTFE balls (Redhill, UK). The tests were performed at 5, 10 and 25 mN applied loads, with 1 cm/s maximum sliding velocity and 0.5 mm half amplitude, in dry conditions and at room temperature. The laboratory temperature and the relative humidity varied between 23 and 26 °C and 40% and 56%, respectively. To be able to perform the tests with the testing parameters ranges used, two medium load (ML) cantilevers with different stiffnesses were used. A ML1 cantilever was used for 5 mN applied load, while a ML2 cantilever was selected for the tests performed at 10 and 25 mN applied loads. The tests were performed with the sliding direction both parallel and perpendicular to the LIPSS direction. Their duration varied between 50 and 1000 sliding cycles (corresponding to about 15 to 450 s). At least three tests were performed for each set of conditions, in different regions of the specimen. Polished samples were tested under the same conditions for comparative purposes (reference samples). The evolution of the wear scars surface morphology was studied by scanning electron microscopy (SEM). Cross-sections of the specimens tested with 1000 cycles in the parallel sliding direction prepared by ion milling were also examined by SEM. Semi quantitative element analysis of the wear surfaces was performed by energy-dispersive X-ray spectroscopy (EDS,) using an EDS attachment to the FEG-SEM.

## 3. Results

### 3.1. Laser-Processed Surfaces

**Figure 1:**
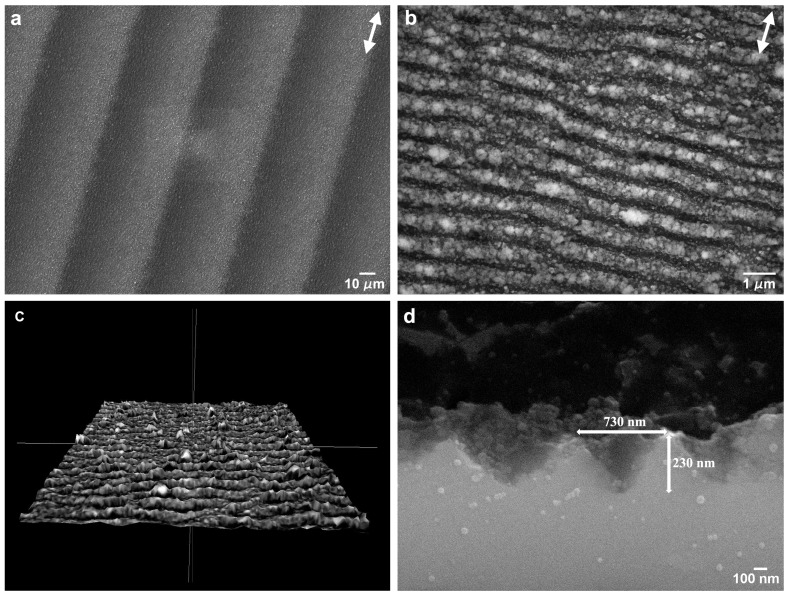
(**a**) SEM micrograph of a silicon surface textured with laser-induced periodic surface structures (LIPSS); (**b**) higher magnification image; (**c**) 3D surface reconstruction of the textured surface; (**d**) SEM micrograph of the cross-section of the LIPSS. The double arrows in (**a**,**b**) indicate the beam polarization direction.

The SEM micrograph in Figure 1a,b illustrates the surface textures created by the femtosecond laser treatment. Figure 1c shows a 3D reconstruction of the surface and Figure 1d a SEM micrograph of the cross-section perpendicular to the LIPSS direction. Due to the Gaussian profile of the laser beam, the laser tracks are slightly deeper in the centre, creating a surface waviness with a period of approximately 50 µm and a depth of 200 nm when consecutive tracks are overlapped (Figure 1a). The LIPSS have approximately 730 nm average period and 230 nm height, as measured by analysis of stereoscopic pairs and in good agreement with literature [51]. The LIPSS form in the direction perpendicular to that of the laser beam polarization, indicated in Figure 1a by the double arrows. Their peaks are covered with ablation debris (Figure 1b), which can be removed by ultrasonic cleaning. The arithmetic roughness of the surface, Ra, is approximately 0.77 µm and its fractal dimension is approximately 2.1, obtained from the analysis of stereoscopic pairs. This topography corresponds to the formation of low-spatial frequency laser-induced periodic surface structures [9].

### 3.2. Tribological Tests

**Figure 2:**
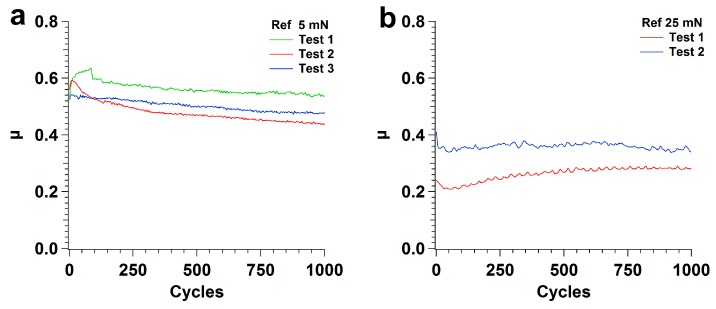
Variation of the friction coefficient with the number of cycles (proportional to the sliding distance) of consecutive tests for the polished Si specimen (Ref = Reference sample) at (**a**) 5 mN and (**b**) 25 mN.

Figure 2 illustrates the variation of the friction coefficient during three consecutive tests performed on different regions of polished Si specimens (reference samples) at 5 and 25 mN applied loads. Since the tribological tests were performed in linear reciprocating motion, the sliding distance is proportional to the number of cycles. The tests performed on polished specimens with 10 mN loads presented non-reproducible stick-slip behavior due to the cantilever, and the corresponding results were not included in the figure. Independently of the testing conditions, some dispersion of the friction coefficient values is observed, due to surface irregularities of the PTFE counterbodies. At 5 mN (Figure 2a), a short (<50 cycles) run-in period was observed during which the friction coefficient increased. After the run-in period, the friction coefficient decreased steadily from 0.60 to 0.54, 0.52 to 0.44 and 0.52 to 0.48 (between 100 and 1000 cycles for tests 1, 2 and 3, respectively). The average friction coefficient between 750 and 1000 cycles was 0.49 ± 0.05. At 25 mN (Figure 2b), after a run-in period lasting about 50 cycles during which the friction coefficient decreased, a slight but steady increase of the friction coefficient with the sliding distance was observed, eventually reaching a steady state after 750 cycles. The average value of the friction coefficient between 750 and 1000 cycles was 0.32 ± 0.05.

**Figure 3:**
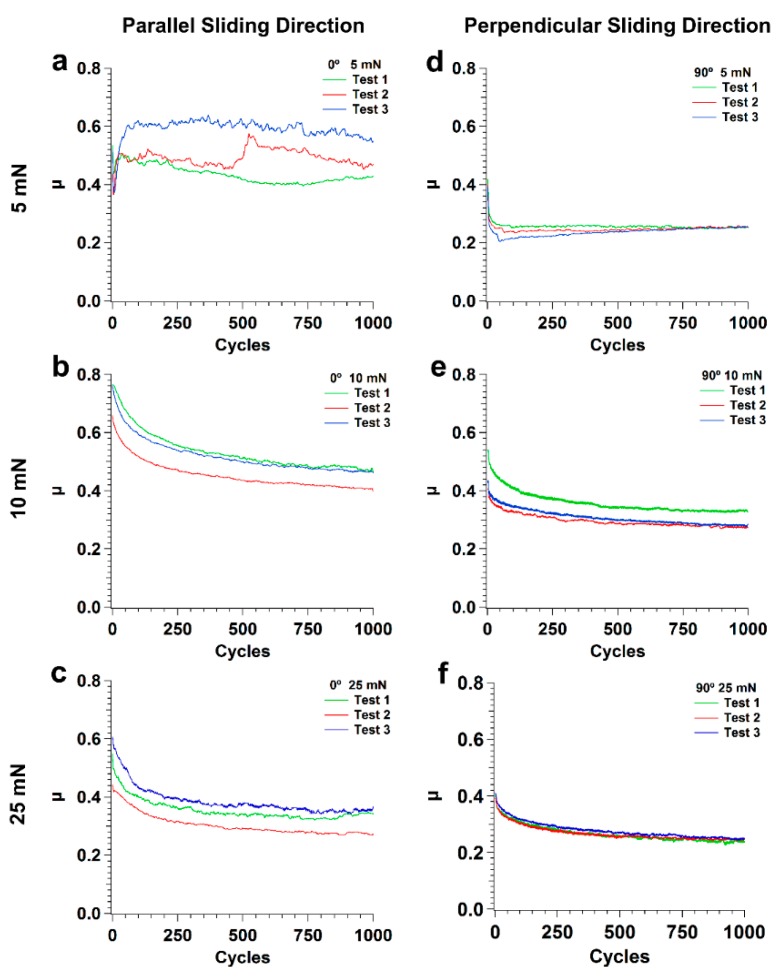
Variation of the friction coefficient with the number of cycles (proportional to the sliding distance) of three consecutive tests performed on textured specimen sliding in the parallel (0°) direction at (**a**) 5, (**b**) 10 and (**c**) 25 mN, and in the perpendicular (90°) direction at (**d**) 5, (**e**) 10 and (**f**) 25 mN.

The friction coefficient results obtained for the textured specimens are plotted in Figure 3. For tests at 5 mN in the parallel sliding direction there was poor reproducibility, for reasons that will be discussed later. For this applied load, the average value of the friction coefficient between 750 and 1000 cycles was 0.49 ± 0.08. For the tests at 10 and 25 mN in the parallel sliding direction, the reproducibility of the results was much better (Figure 3b,c). The friction coefficient decreased continuously since the beginning of the test, initially (0–50, 100 cycles) faster, and no run-in period could be distinguished. The average values of the friction coefficient between 750 and 1000 cycles, were 0.46 ± 0.04 and 0.35 ± 0.02 for 10 and 25 mN, respectively.

For the perpendicular sliding direction and an applied load of 5 mN (Figure 3d), after a short run-in period during which the friction coefficient decreased sharply, its value remained constant within the limits of the experimental error. The average value of the friction coefficient in the interval 750–1000 cycles was similar for in all tests, 0.25 ± 0.01. For 10 and 25 mN (Figure 3e,f), the friction coefficient decreased steadily from the beginning of the tests, reaching average values of 0.30 ± 0.03 and 0.25 ± 0.01 in the range 750–1000 cycles, respectively.

**Figure 4:**
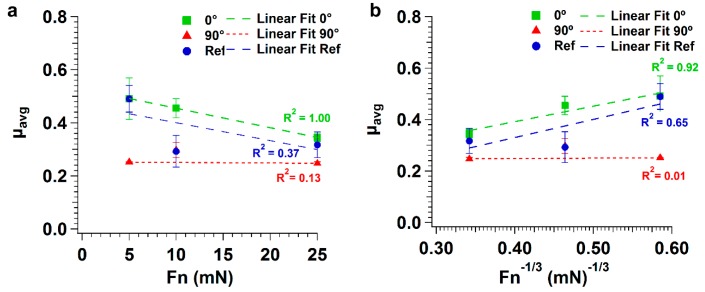
Variation of the average friction coefficient with (**a**) applied load and (**b**) with the applied load to the power (−1/3), for polished (Ref = reference sample) and textured specimens, in the parallel (0°) and perpendicular (90°) sliding directions.

The variation of the average friction coefficient in the interval 750–1000 cycles with applied load is illustrated in Figure 4a. The friction coefficient decreased with the applied load for both polished and textured specimens when sliding in the parallel direction, but for the perpendicular sliding direction the variation was negligible. The lowest values of the friction coefficient were obtained for the perpendicular sliding direction, and the highest for the parallel sliding direction. The difference decreased with increasing load.

The three data sets were linearly fit by the least squares method for polished and textured specimens. The fitting was extremely good for the textured specimens in the parallel sliding direction (R^2^ = 1.00), but less so for the polished specimens and the textured specimens tested in the perpendicular sliding direction (R^2^ = 0.37 and 0.13, respectively). In case of elastic contact, the Hertz theory predicts that the real contact area was proportional to (load)^2/3^ and thus that the friction coefficient would be proportional to (load)^−1/3^ [52]. If the average friction coefficient was fitted with a (load)^-1/3^ function the correlation was weaker for the textured specimens (R^2^ = 0.92 and R^2^ = 0.01 for the parallel and perpendicular sliding directions, respectively), as illustrated in Figure 4b, and better for the polished specimens (R^2^ = 0.65).

### 3.3. Surface Morphology

**Figure 5:**
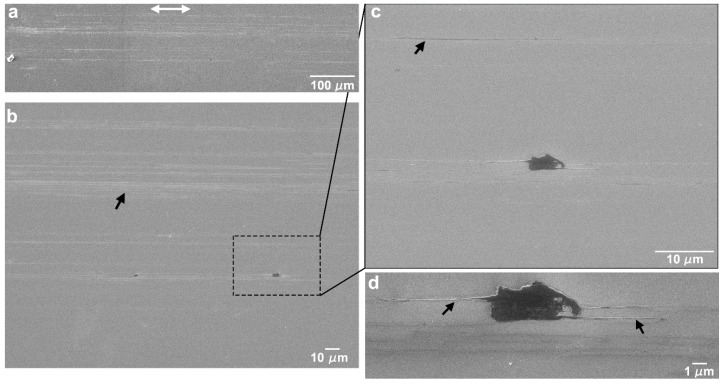
SEM micrographs of the centre of wear tracks on polished Si specimen performed at 5 mN. (**a**,**b**) General view of the wear track at different magnifications. (**c**) Transferred PTFE material. (**d**) Detailed view of the transferred PTFE material. The double arrow indicates the sliding direction.

**Figure 6:**
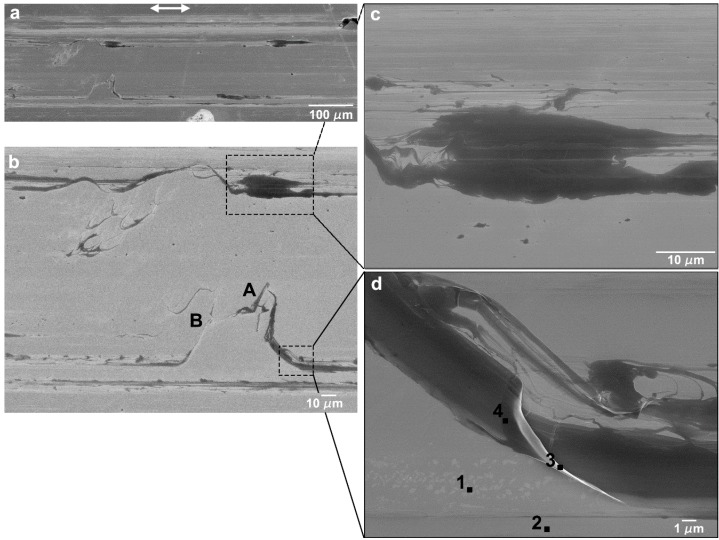
SEM micrographs of the centre of wear tracks on the polished Si specimen performed at 25 mN. (**a**,**b**) General views of the wear track at different magnifications. (**c**) Detail of the transferred PTFE lump. (**d**) Loosely adherent PTFE film. The double arrow indicates the sliding direction.

**Figure 7:**
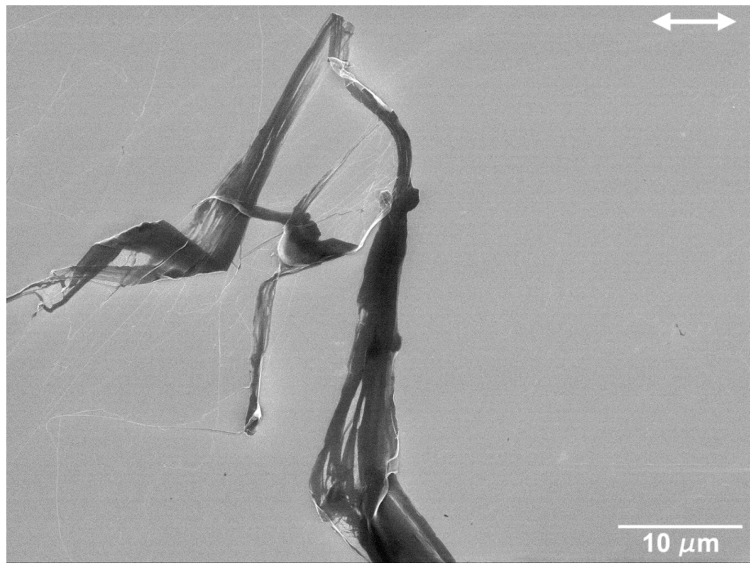
PTFE fibers drawn from PTFE ribbons in zone A of the wear track produced at 25 mN illustrated in Figure 6b.

**Figure 8:**
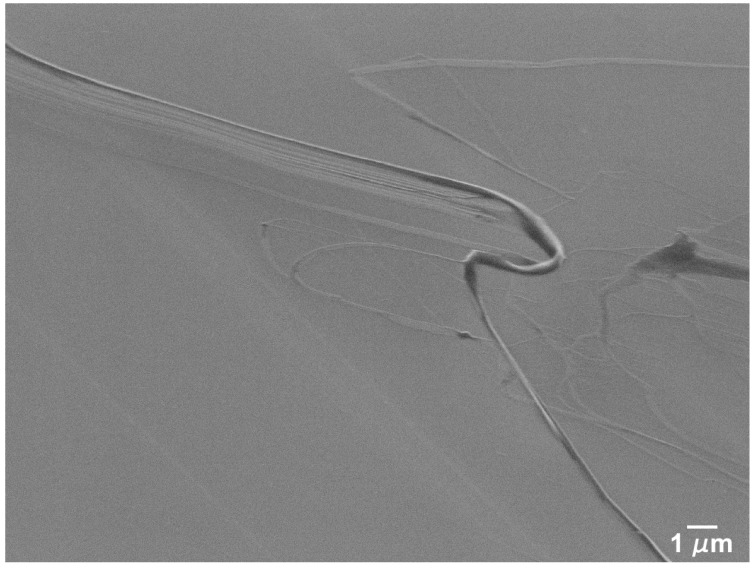
PTFE fibers drawn from a PTFE ribbon joining to form a thin film in zone B of the wear track produced at 25 mN illustrated in Figure 6b, taken at 45° tilt.

SEM micrographs of the worn surfaces in the polished Si specimens are presented in Figure 5, Figure 6, Figure 7 and Figure 8. It can be observed that a transfer layer of PTFE formed at the specimen surface for all applied loads. For 5 mN (Figure 5), the transferred material took the form of thin PTFE films with a fibrous structure (observed in the region indicated by the black arrow in Figure 5b). The dark regions observed in Figure 5c,d corresponded to thick lumps of PTFE. Ribbons a few microns wide (indicated by the black arrow) were drawn from these lumps of material, a process that was previously observed by Makinson and Tabor in similar testing conditions [35]. The surface morphology of wear tracks produced at 25 mN are presented in Figure 6. For these testing conditions, the transferred material was more irregularly distributed and presented a wider range of morphologies (Figure 6a). PTFE lumps and ribbons were observed, but thin films appeared in some areas as well (Figure 6b). Figure 6c shows PTFE ribbons drawn from a lump of transferred material and the fibers formed by the disaggregation of the ribbons. Ribbons usually form when PTFE lumps are rolled or twisted between the counterbody and the specimen surface [35]. They are formed by an arrangement of parallel fibers, which can be seen in the figure connecting the ribbons and the thin films. The lumps of PTFE consist of overlapped PTFE layers drawn at consecutive scans of the counterbody over the specimen surface. This is consistent with the mild stick-slip observed in the friction curves, independently of the load. On the other hand, the periodic adhesion between the transfer layer and the PTFE counterbody may also explain the large stick-slip oscillations observed for polished specimens and 10 mN applied load. In some areas the PTFE films detached from the surface, as illustrated in Figure 6d. In other regions, the film is smudged, indicating that it was dragged by the counterbody in its motion (Figure 6b). The relation between the PTFE fibers and the ribbons is illustrated in Figure 7 and Figure 8, corresponding, respectively, to the zones indicated by A and B in Figure 6b. In Figure 7, fibers can be seen at the edges of a PTFE ribbon. Figure 8 depicts a SEM micrograph taken at a 45° tilt, that shows PTFE fibers drawn from the ribbons. The fibers were a few tens of nanometers wide and agglomerate to form films a few hundreds of nm wide. The area covered by the transferred PTFE film and the width of the wear tracks increased with increasing load.

The results of the EDS analysis performed on the wear tracks surfaces were only semiquantitative, because the electrons of the incident electron beam penetrated the material to a depth larger than the thickness of the transferred material, so characteristic X-rays were emitted simultaneously from the transferred material and from the substrate. Due to the irregular surface topography and the heterogeneity of the surface, the ZAF correction (atomic number (Z), absorption (A) and fluorescence (F)) necessary for quantification also could not be performed. However, since the Si characteristic X-rays were attenuated by the PTFE layer proportionally to the thickness of this layer, the percentage of Si measured at each point gave an indication of the thickness of the transfer material at that particular location. EDS point analysis was performed at the points indicated by 1 to 4 in Figure 6d, leading to the following results: Points 1 and 2, 100 wt.% Si (no transfer material film), Points 3 and 4, 94 wt.% Si, corresponding to regions covered with a transfer film. This value is typical of areas covered with transferred material, although in some regions where the transfer material thickness is larger, the Si content can be as low as 81 wt. %.

**Figure 9:**
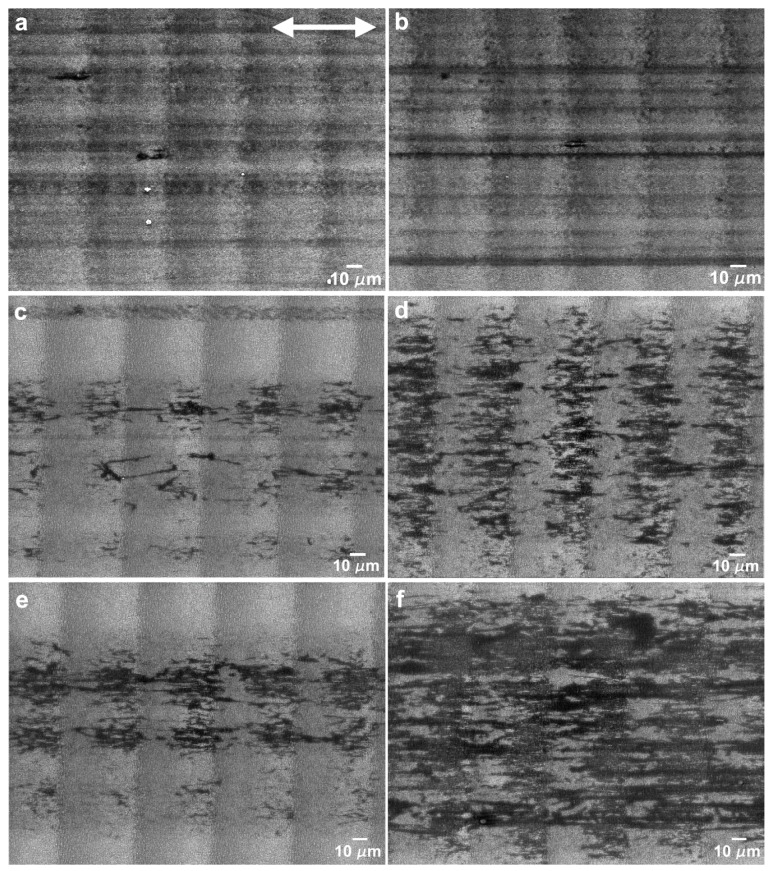
Wear tracks at 5 mN after (**a**) 50 cycles and (**b**) 1000 cycles; at 10 mN after (**c**) 50 cycles and (**d**) 1000 cycles; and at 25 mN after (**e**) 50 cycles and (**f**) 1000 cycles. The double arrow indicates the sliding direction, parallel to the LIPSS orientation.

**Figure 10:**
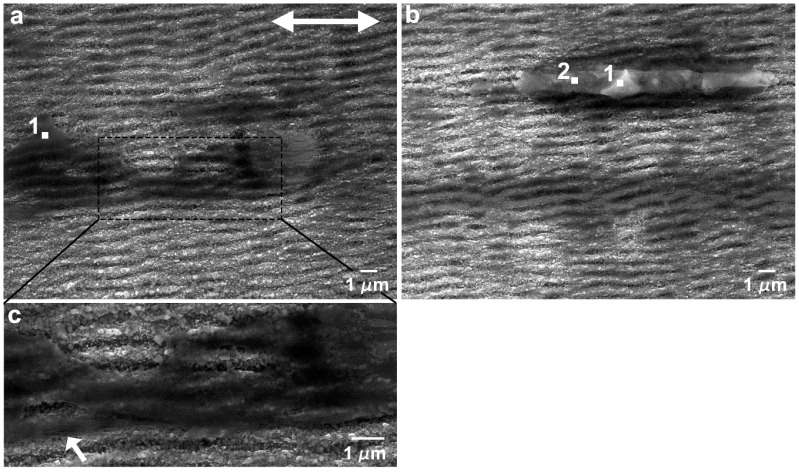
SEM micrographs of the wear tracks of the textured specimen in the parallel direction at 5 mN, after 1000 cycles, showing (**a**) PTFE transferred film and (**b**) worn LIPSS and PTFE roll. (**c**) Detailed view of (**a**), illustrating the fibrous nature of the PTFE film. The double arrow indicates the sliding direction.

**Figure 11:**
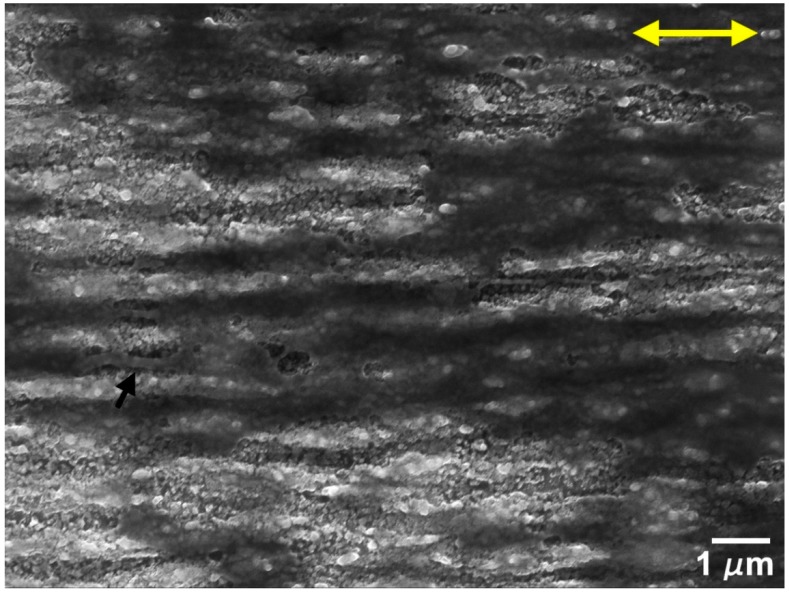
SEM micrograph of a wear track produced at 10 mN and 400 cycles. The double arrow indicates the sliding direction.

**Figure 12:**
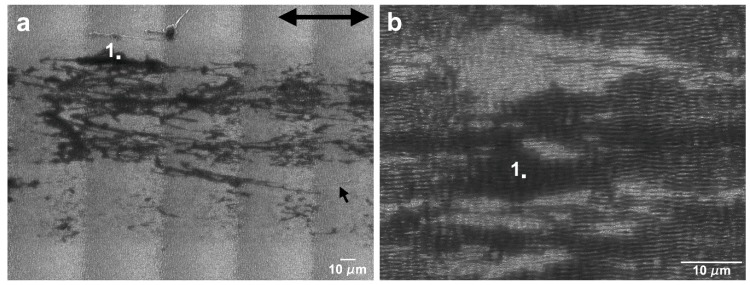
Energy-dispersive X-ray spectrometry (EDS) point analysis on the wear tracks produced with 25 mN load and (**a**) 50 cycles and (**b**) 1000 cycles. The double arrow indicates the sliding direction.

**Figure 13:**
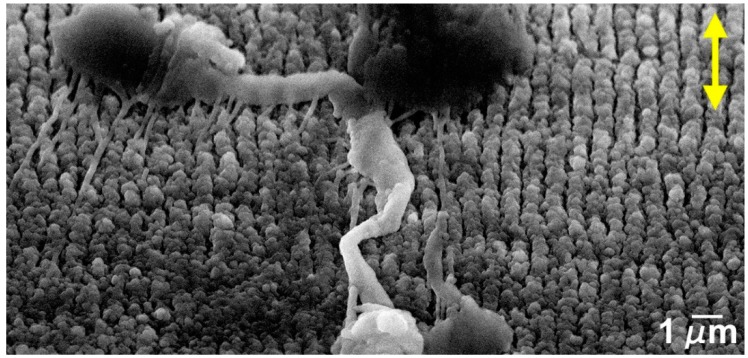
SEM micrograph of the cross-section of a wear track taken at 45° tilt. The double arrow indicates the sliding direction.

The morphology of the wear tracks created in the parallel sliding direction is depicted in the SEM micrographs of Figure 9. The wear tracks obtained with a load of 5 mN (Figure 9a,b, 50 and 1000 cycles, respectively) were covered with stripes of a transferred PTFE film, aligned with the sliding direction, which filled the valleys between the LIPSS crests, seen as darker regions in the SEM images. In some regions, thicker PTFE layers were observed (seen in more detail in Figure 10a), which sometimes were rolled between the two sliding surfaces to form twisted rolls (Figure 10b). The fibrous structure of the PTFE film was clearly observed in Figure 10c (indicated by the arrow). In the longer tests (1000 cycles), the LIPSS crests were worn out in the central region of the tracks (Figure 10b). For this applied load, the area covered by PTFE did not vary significantly with the number of cycles, as shown by comparing Figure 9a,b, but the thickness of the PTFE stripes increased, as demonstrated by the larger contrast of Figure 10b as compared to Figure 10a. For 10 mN (Figure 9c,d), the morphology of the transferred material was different. It consisted mainly of lumps, thick layers and ribbons, filling the spaces between the LIPSS, as shown in the region indicated by the black arrow in Figure 11. The transferred material did not form continuous stripes but concentrated at the elevations of the surface waviness (Figure 9c). The area covered with PTFE increased with the sliding distance (as shown by comparing Figure 9c,d), but the wear tracks were never uniformly covered with PTFE. The morphology of the transferred material for 25 mN was similar, but the area covered with transferred material was already important after 50 cycles (Figure 9e). This area increased with the duration of the tests (Figure 9f), but even after 1000 cycles PTFE-free regions were still observed in the depressions of the surface waviness (Figure 9f). Similarly, to what happens for 10 mN, the transferred material took the form of ribbons (shown by an arrow in Figure 12a), sheets and lumps of different thicknesses (Figure 9f and Figure 12b). A SEM micrograph of a cross-section of a wear track (Figure 13) taken at 45° tilt showed that the transfer material filled the depressions between the LIPSS. This figure also shows how the PTFE lumps and ribbons attach to the surface through filaments of the same material.

The differences in the thickness of the transferred layer from region to region were reflected in the results of the EDS analysis. The EDS analysis at point 1 in Figure 10a led to 73 wt. % Si, and at points 1 and 2 on the PTFE roll in Figure 9b to 78% Si and 72% Si, respectively, corresponding to a moderately thick layer of transferred material. For 25 mN at 50 and 1000 cycles the layers of transferred material were thicker: 49 wt. % Si was measured in point 1 of Figure 12a; while in point 1 of Figure 12b the Si content was 69 wt.%.

**Figure 14:**
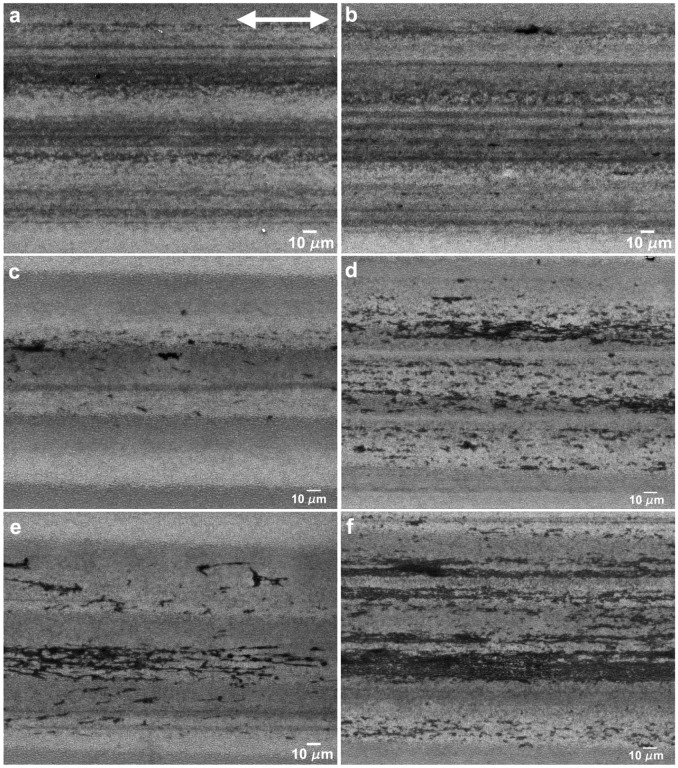
SEM micrographs of the central region of wear tracks produced in the perpendicular sliding direction at 5 mN after (**a**) 50 cycles and (**b**) 1000 cycles; at 10 mN after (**c**) 50 cycles and (**d**) 1000 cycles and at 25 mN after (**e**) 50 cycles and (**f**) 1000 cycles. The double arrow indicates the sliding direction.

**Figure 15:**
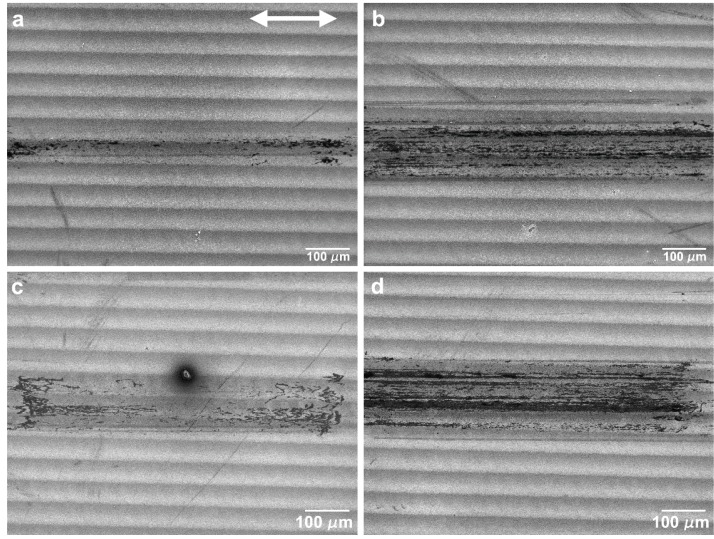
SEM micrographs of the wear tracks of the textured specimen in the perpendicular direction at 10 mN (**a**) after 50 cycles and (**b**) 1000 cycles and at 25 mN (**c**) after 50 cycles and (**d**) 1000 cycles. The double arrow indicates the sliding direction.

**Figure 16:**
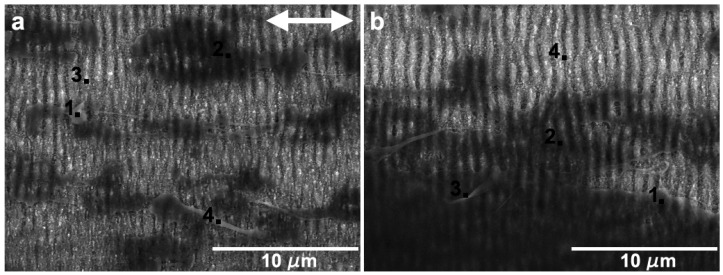
EDS point analysis on wear tracks performed with (**a**) 1000 cycles at 10 mN cycles and (**b**) 400 cycles at 25 mN. The double arrow indicates the sliding direction.

The evolution of the wear track morphology for the tests performed in the perpendicular sliding direction was analogous to the parallel sliding direction, with some differences (Figure 14 and Figure 15). As for the parallel sliding direction, for 5 mN and 50 cycles (Figure 14a) the wear track presented stripes of a PTFE transfer film (darker in the SEM images) and a few areas where the PTFE transfer layer was sufficiently thick to cover the LIPSS. The area covered with PTFE did not change significantly with the number of cycles (compare Figure 14a,b). At higher loads (10 and 25 mN), the areas covered with thick PTFE layers were larger and in greater number, but they remained discontinuous, except after 1000 cycles at 25 mN. For 10 mN, the LIPSS peaks showed traces of wear in the central region of the track since 50 cycles, and the PTFE transfer film and lumps concentrated at the tracks periphery (Figure 15a). The area covered with PTFE increased with increasing sliding distance, but it did not cover entirely the wear tracks, even after 1000 cycles (Figure 14d). Contrarily to what happens in the parallel sliding direction, the transfer film did not fill the space between the LIPSS (Figure 16a) and was covered in some areas with PTFE rolls and ribbons. The evolution for 25 mN was similar, but faster. The transferred material took mainly the form of lumps and ribbons (Figure 16b). After 50 cycles (Figure 14e), a transfer film formed at the periphery of the wear tracks (Figure 15c), which extended to the centre with an increasing number of cycles (compare Figure 15c,d). After 1000 cycles, the transfer layer covered a significant proportion of the wear track surface, but not completely (Figure 14f). It extended to the entire length of the tracks in some regions, contrarily to what happened in the parallel sliding direction, where the transfer material layer was interrupted due to the surface waviness.

Figure 16a,b present the locations of EDS analysis, which led to the following results: (a) The points 1 to 4 (Figure 16a), corresponding to a PTFE ribbon, a PTFE film, the exposed specimen surface and PTFE fibers presented 43 wt.% Si, 77 wt.% Si, 93 wt.% Si and 64 wt.% Si, respectively. The points indicated by 1 to 3 in Figure 16b corresponded to different regions of the PTFE film, and led to 77 wt. % Si (Points 1 and 2) and 73 wt. % Si in Point 3. Point 4 corresponded to the exposed surface and led to 98 wt. % Si and 2 wt. % O.

## 4. Discussion

In order to better understand the friction behavior of textured surfaces sliding against PTFE, it is necessary to take into consideration the morphology of the transferred material, and the deformation and fracture behavior of this polymer. PTFE is a semi-crystalline polymer, which undergoes several phase transformations up to its melting temperature [53]. The low temperature phase (usually designated phase II) presents a triclinic structure and is stable up to 19 °C. At this temperature, it transforms to phase IV, which presents a partially ordered hexagonal structure and is stable between 19 and 30 °C. The high temperature phase is less well-known but is characterized by a higher degree of disorder. Above 150 °C the material becomes amorphous [54]. As expected from these phase transitions, PTFE presents low ductility at low temperature, but the ductility increases considerably with temperature, while its tensile strength decreases, reaching a value of ~5 MPa at about 200 °C [46,54]. The fracture mechanism depends on temperature as well, a brittle behavior being observed below 19 °C and a ductile behavior, with considerable plasticity, above 30 °C [55]. The strength and ductility depend on the strain rate, that decreases with decreasing plasticity while the yield stress increases [56]. Between 25 and 150 °C, the true rupture stress decreases by one half [57] and the crack propagation is accompanied by the formation of fibrils, which provides an effective mechanism to dissipate energy. These mechanical properties have a direct impact on the friction behavior of PTFE. When sliding against highly polished surfaces of materials such as glass [35] and silicon oxide [58] at low sliding speeds (≤1 mm/s) and relatively high temperatures, a very thin film (10–40 nm) of PTFE is transferred to the countersurface, leading to an extremely low friction coefficient at room temperature (~0.04). These films are crystalline and highly ordered, even more than the bulk PTFE polymer, and form when PTFE strands consecutively attach to the substrate and are pulled off from the bulk material to yield an array of parallel fibers [59]. This film presents very low shear strength and, consequently, a very low friction coefficient, making thin PTFE films a highly effective solid-state lubricant. For higher sliding speeds, especially at low temperatures, the material presents a brittle behavior [60] and fractures randomly in irregular particles, which adhere to the countersurface [35]. These particles are then laminated between the two bodies to form films of PTFE with a fibrous morphology, typical of PTFE deformed at moderately high temperatures [60]. The friction coefficient is much higher than 0.3 [35].

The tests performed on polished silicon specimens in the present work lead to values of the friction coefficient of about 0.6, for an applied load of 5 mN, decreasing to ~0.5 when the number of cycles increases, and roughly constant values of 0.3–0.35 at 25 mN (Figure 2). This regime is preceded by a run-in period, during which the friction coefficient either increases (5 mN) or decreases (25 mN) and these variations can be explained by the initial reduction in surface roughness [61] as well as the growth of a low shear strength PTFE transfer film in some areas of the surface. The transfer of PTFE to the silicon surface occurs due to the large adhesion forces between the Si substrate and the PTFE (mainly van der Waals and electrostatic forces). Since, for the scanning speed used, deformation is occurring in the PTFE low plasticity regime, it fractures, forming particles that adhere to the Si surface and are deformed between the contact surfaces to form the transfer layers observed in Figure 4 and Figure 5 [32,52,62,63]. After the run-in, the friction coefficient decreases progressively as the areas covered with PTFE expand gradually, preventing further pulling-off of PTFE particles. However, its value remains high (>0.4) because the transfer layer is thick and irregular due to the high sliding speed used (10 mm/s). This is confirmed by the SEM micrographs of Figure 5. On the other hand, 1000 cycles were not enough for the PTFE transfer film to cover completely the Si surface, so the friction coefficient did not reach a steady state (Figure 2a). For 25 mN, the overall value of the friction coefficient was lower than for 5 mN. The friction coefficient decreased slightly during the run-in period (Figure 2b), as the PTFE transfer layers begin forming on the Si surface [38]. This evolution was faster than for 5 mN and the higher interface shear stress might lead to a more perfect alignment of the PTFE chains parallel to the sliding direction, thus explaining the lower value of friction coefficient obtained as compared to that at 5 mN. Periodic oscillations of the friction force are observed indicating that stick-slip occurred during testing. Comparative tests performed with different cantilevers showed that the presence and amount of stick-slip depend on the cantilever and on the applied load, which explains the poor reproducibility of the results obtained for certain testing conditions (in particular, 10 mN). It is well known that the tangential contact stiffness determines the slope in the stick stage, while the stick-slip amplitude is determined by the cohesive strength and surface energy of the sliding interface [63,64,65]. The tests were performed with a ML1 cantilever (a soft cantilever), which revealed itself adequate for 5 mN, but too soft for 10 and 25 mN. On the other hand, the stiffer ML2 cantilever was adequate for 25 mN, but too stiff for 5 and 10 mN, and led to stick-slip, particularly for 5 mN applied load. No stick-slip was observed for the textured specimens, despite using the same cantilevers. It is well-known that the force required to overcome adhesion increases with increasing contact area. Since texturing reduces the contact area, adhesion is sufficiently reduced to avoid stiction. The average value of the friction coefficient in the steady state (after 750 cycles) is 0.32, similar to the value reported by Makinson and Tabor [35] for PTFE sliding on glass in the high friction regime (>0.33). This value is expectable taking into consideration that, for the relatively high sliding velocity used in the present work (10 mm/s), PTFE must present a brittle behavior, with fracture and lump formation, accompanied by a large material displacement [35,63]. However, due to the stick-slip phenomenon, the transfer of PTFE to the Si surface is discontinuous. The regions where the transfer layer was thicker correspond to the stick (static) regions, where adhesion and, consequently, the friction coefficient were larger and the deformation of the polymer and the amount of transferred material were more important. In the slip region, PTFE was transferred to the counterbody more smoothly, in the form of a thinner fibrous film. In the slip stage, the interface shear strength was lower, causing the friction coefficient to decrease momentarily, until the moment when adhesion was re-established. The stick-slip regime was maintained during the entire test, so consecutive layers of PTFE were transferred to the same regions of the surface, building the thick multilayer transfer of polymer shown in Figure 6c. Since stick-slip was milder at 25 mN, this effect was less marked. The increase of the friction coefficient during the tests was due to the shearing and ripping out of the PTFE film from the Si surface, as shown in Figure 7 [34].

The friction coefficient of PTFE sliding on polished silicon decreased with increasing applied load (Figure 4), in good agreement with the literature [32,66]. This evolution is often observed for polymers, including PTFE [38,52,67]. According to the SEM observations (Figure 5 and Figure 6), the area occupied by the transferred PTFE films increased with applied load, which in turn decreased the interfacial shear strength and, consequently, the friction force [66,68]. The friction behavior observed for polished silicon was similar to that previously observed by other authors for PTFE sliding on glass [35,38,63]. The present results also agreed with the conclusions of Blanchet and Kennedy [34] who found that the critical velocity for the mild-to-severe wear transition for PTFE sliding on stainless steel under an average contact pressure of 6.55 MPa was 0.8 cm/s, slightly lower than the sliding velocity used in our work.

The textures observed in Figure 1 consisted of low-spatial frequency laser-induced periodic surface structures (LSFL) [6]. The formation of these structures is believed to be due to the modulation of the absorbed radiation intensity created by the interference of the incoming laser beam with scattered radiation propagating parallel to the solid surface [14] or with surface plasmon polaritons excited by the laser beam [69]. The LIPSS peaks were covered with ablation debris. This could be explained by the fact that, when the predominant ablation mechanism at the absorbed laser intensity maxima is liquid spallation [15], the shallow liquid layer created by the laser beam is fragmented into a large number of extremely small droplets, which are expelled in the confined space between the LIPSS and partially redeposit on their peaks. As shown by these authors [15], the only phase transformation introduced in the surface layer of material by this surface treatment is melting of a layer of material with a thickness varying in the 65–130 nm range. The laser treatment also induces the formation of defects in a layer of material less than 1 µm deep, as shown by Sedao et al. [70], but these defects do not seem enough to change significantly the surface hardness of the material [71], so the observed effects are mainly to be accounted by the surface topography.

The presence of the LIPSS texture changes significantly the tribological behavior of the system. Firstly, the friction coefficient decreased during the run-in period, as the PTFE chains align with the sliding direction and the polymer starts being transferred to the Si surface, reducing adhesion and the surface roughness [62]. After the run-in period, the friction coefficient continued to decrease, in particular for the higher loads. The tests performed in the parallel sliding direction with 5 mN applied load presented low reproducibility. The PTFE transfer film was not uniform and regularly distributed over the wear track surface (Figure 9a,b). In some regions the transfer layer was sufficiently thick to cover the LIPSS and PTFE ribbons were drawn out from the PTFE layer (Figure 9a). On the other hand, the wear particles that were trapped between the two surfaces were rolled and took a cylindrical shape (Figure 9b). This complex morphology indicated that the PTFE counterbody was fracturing randomly and a range of complex interactions occurred during the tests, which explain the dispersion of the friction coefficient value and its abrupt variations observed in the tests. For 10 and 25 mN, instead of a thin PTFE film, a thick transfer layer formed, initially only at the elevations of the surface waviness caused by the overlap of consecutive laser tracks (Figure 9c,e). The relative area occupied by these layers increased progressively with the number of cycles, explaining the decrease in friction coefficient, but the wear track was never completely covered even after 1000 cycles (Figure 9f). As shown in Figure 13, the space between the LIPSS was occupied by the PTFE fibers, which anchor the PTFE transfer layers to the Si substrate.

When testing in the perpendicular sliding direction at 5 mN load, the friction coefficient remained almost constant after the run-in period, with an average value of 0.25, because the proportion of the wear track surface covered with PTFE did not increase significantly with the number of cycles. The transfer layer occupied a larger area, was more uniformly distributed and large PTFE particles were less frequent than for the parallel sliding direction (Figure 14a,b). Consequently, the friction curves were smoother and more reproducible. For 10 and 25 mN, the friction coefficient decreased continuously, approaching the steady state after 1000 cycles. At these loads, the PTFE film initially formed at the periphery of the wear track (Figure 15a,c), where the contact pressure was lower, then extended to the centre of the wear track, occupying preferentially the elevations of the surface waviness, which were now parallel to the sliding direction, but eventually covered the wear track almost completely (Figure 15b and d at 10 and 25 mN, respectively). Its distribution was more uniform than in the parallel sliding direction (Figure 15b,d). The fraction of the wear tracks covered with PTFE increased with the number of cycles, causing the slight decrease in the friction coefficient observed.

In order to explain the observed variation of PTFE/Si tribological behavior with the testing parameters, its mechanical properties must be taken into consideration. As the applied load increased, there was a monotonic increase of the interface temperature, which favored the transition of PTFE to less orderly crystalline phases (Phase I, above 30 °C) and led to a steady decrease in the strain to failure rate [57]. This decrease in PTFE ductility facilitated plastic deformation and, consequently, the formation of the PTFE transfer film at the Si surface.

In order to explain the observed behavior for polished and textured specimens, one must compare the contributions of adhesion and elastic deformation to friction in both cases. The relative contributions of adhesion and elastic deformation of asperities in the case of the textured specimens could be evaluated for the perpendicular sliding direction by estimating the elastic energy stored in the deformation field of the polymer, $U_{elastic}$, and the gain in adhesion energy, $U_{adh},$ by using the following equations [72]:

(1) $$U_{elastic}=E\lambda h^{2},$$

(2) $$U_{adh}=\Delta \gamma \lambda^{2},$$

where E is the elastic modulus of PTFE, h and λ are the height and width of the substrate’s cavity occupied by the polymer, respectively and Δγ is the change in surface free energy per unit area. Considering that the height and the width of the LIPSS were about 200 and 400 nm, respectively, then $U_{elastic}\;\mathrm{was}\;\sim 3.68\;pJ$ and $U_{adhesion}\;\mathrm{was}\;\sim 0.04\;pJ$, indicating that the elastic contribution to friction was more significant than that of adhesion. For the parallel sliding direction, λ can be estimated as 6 µm, which is about 1/4 to 1/5 of the Hertzian radius of contact at 5 to 25 mN, and $U_{elastic}\;\mathrm{as}\;\sim 110\;pJ$ and $U_{adhesion}\;\mathrm{as}\;\sim 36\;pJ$, which is more than two orders of magnitude larger than for the perpendicular sliding direction. The same estimates made for polished silicon lead to $U_{elastic}\;\mathrm{as}\;\sim 46\;pJ$ and $U_{adhesion}\;\mathrm{as}\;\sim 100\;pJ$, assuming that for smooth surfaces $\frac{h}{\lambda }\;\sim 0.01$ or smaller [72]. From these estimates alone, we could conclude that the contribution of adhesion is large for the polished specimens, but much smaller for the textured specimens. Thus, a lower friction coefficient is expected for the textured specimens. In fact, the friction coefficient of the textured specimens tested in the parallel sliding direction is very similar to the value obtained for polished specimens, while the value for the perpendicular direction, under similar testing parameters, is lower (0.46 and 0.35 for 10 and 25 mN as compared to 0.30 and 0.25; Figure 4). Texturing increases the surface roughness, favoring plastic instead of elastic contacts, and increasing the ploughing component of friction. This is supported by the fact that, while the average friction coefficient of the polished specimens shows good correlation with the applied load^−1/3^, indicating a predominantly elastic contact, the average friction coefficient measured in the textured specimens shows a better correlation to the applied load, suggesting a transition to a predominantly plastic contact regime [64]. In the elastic contact regime, an increase of the surface roughness leads to a decrease of the real contact area and, consequently, of the adhesion contribution to friction. This increase of roughness may also lead to an increase of the plastic deformation and a shift in the wear regime from sliding wear to abrasive wear, affecting mainly the softer material. Thus, the presence of LIPSS may change the PTFE/Si wear mechanism, increasing the contribution of plastic deformation (ploughing and cutting) to the friction [64]. Due to this change in the wear mechanism, the proportion of PTFE transferred lumps is usually larger for the textured specimens, particularly at high loads.

## 5. Conclusions

1. Tests performed on polished Si specimens show that the friction coefficient decreased steadily with testing time for 5 mN, with an average value of 0.5, and it increased slightly with testing time for 25 mN, with an average value of 0.3. At 5 mN, the wear tracks were characterized by the formation of very thin PTFE films with a fibrous structure and of thicker layers of PTFE, from which ribbons, a few microns wide, were drawn. Due to the relatively high sliding speed used and the fact that PTFE was in the low plasticity regime, the PTFE film was generally thick and irregular, explaining the particularly high value of the friction coefficient at this load (0.5). The wear track area covered by PTFE increased with testing time, preventing further pulling-off of PTFE particles and causing the friction coefficient to decrease progressively. The wear track never completely covered the Si surface, and so the friction coefficient did not reach steady state. At 25 mN, the wear track showed similar elements (lumps, ribbons and thin film), but, despite these forming faster than at 5 mN, the wear track was never completely covered with PTFE due to the presence of mild stick-slip, which prevented the formation of a uniform film over the Si surface. Due to this discontinuous material transfer, the friction coefficient increased during the tests, as the PTFE film was sheared and ripped-out from the Si surface. The higher interface shear stress facilitated the alignment of the PTFE chains parallel to the sliding direction, explaining the lower friction coefficient values in comparison to the 5 mN load. Such a decrease of the friction coefficient with load could be explained by the increase in area covered with transferred PTFE with increasing load, which in turn decreased the interfacial shear strength and, consequently, the friction force.
2. Tests performed with similar parameters in textured specimens showed that LIPSS changed significantly the tribological behavior of this system. Overall, the friction coefficient decreased with testing time in both sliding directions, more significantly for higher applied loads, except at 5 mN in the parallel sliding direction where random fracture of the PTFE counterbody occurred. This decrease was due to the increase in the area occupied by PTFE, which was further facilitated at higher loads due to the increase in interface temperature and consequent decrease in PTFE ductility. For the parallel sliding direction, the transfer film tended to be thick and formed initially only at the elevations of the surface waviness caused by the overlap of consecutive laser tracks. It progressively expanded, but the wear track was never completely covered. For all applied loads, the PTFE fibers occupied the space between the LIPSS, anchoring the transfer layers to the Si substrate. For the perpendicular sliding direction, the PTFE film formed initially at the periphery of the wear track, then progressively extended to the centre, while still occupying preferentially the elevations of the surface waviness. After 1000 cycles and at higher loads, the wear tracks were almost completely covered, more uniformly and occupying a larger area than for the parallel sliding direction, explaining the lower friction coefficient obtained at a steady state.
3. Texturing increased the surface roughness, favoring plastic instead of elastic contacts, increasing the ploughing component of friction and shifting the predominant wear regime of PTFE from sliding to abrasive wear. This change in wear mechanisms led to more PTFE transferred lumps in the textured specimens than in the polished ones, particularly at high loads. The orientation of the surface features, namely of the surface waviness, relative to the sliding direction also had an important effect on friction. Abrasion of PTFE was more pronounced when sliding parallel to the LIPSS because the surface waviness was transverse to the sliding direction, increasing further the ploughing contribution to friction in this case. The smaller abrasion and consequent more uniform distribution of the thin PTFE transfer film in the perpendicular sliding direction at all loads was responsible for the overall lowest friction coefficient values.
